# Impact of Depression, Resilience, and Locus of Control on Adjustment of Health-Related Expectations in Aging Individuals With Chronic Illness

**DOI:** 10.3389/fpsyg.2022.867785

**Published:** 2022-04-28

**Authors:** Aline Schönenberg, Hannah M. Zipprich, Ulrike Teschner, Tino Prell

**Affiliations:** ^1^Department of Geriatrics, Halle University Hospital, Halle (Saale), Germany; ^2^Department of Neurology, Jena University Hospital, Jena, Germany

**Keywords:** quality of life, coping, depression, resilience, locus of control, health behavior, older adults

## Abstract

**Objectives:**

Quality of Life (QoL) depends on the discrepancy between desired and current experiences (referred to as the Calman gap), thus in chronic illness, adjustment of expectations and interpretation of the current situation are crucial. Depression is known to influence this gap, and the present study aims to further assess the role of resilience and health locus of control (HLC).

**Methods:**

A total of 94 patients (age *M* = 71.8, SD = 7.7 years) with neurological disorders were screened *via* telephone regarding depression, resilience and HLC. Current and desired state of several life domains were assessed, such as Fitness, General Health, Pain, Daily Activities, Finances, Leisure, and Family. Elastic net regularization and analyses of variance were used to disentangle the impact of depression, resilience, HLC, and sociodemographic factors on the perception of current and desired state, and the gap between both.

**Results:**

A gap was present for all domains but largest for pain. Interpretation of the current state was linked to desired state, HLC, and age. All gaps were related to depression; certain domains were in addition influenced by resilience, HLC and sociodemographic factors. Of note, for most domains, patients did not select the highest possible desired state.

**Conclusion:**

Older patients with neurological disorders report a gap between current and desired state for many aspects of life. Adjusting expectations is beneficial in the face of declining health, but a reasonably increased desired state may positively influence the perception of the current situation. Depression negatively influences the interpretation of the Calman gap.

## Introduction

Quality of Life (QoL) is strongly influenced by the difference between the expectations or wishes of a person, and their current experience (Carr et al., 2001). This discrepancy between the current situation and the ideal, desired state has first been explicitly described by K.C. Calman and is thus referred to as the Calman Gap. The Calman gap spans across an individual’s experience of many different aspects of life (Calman, 1984; Martínez-Martín, 1998; Prell et al., 2020). QoL may vary between people, as it is based on an individuals’ experience and perception, and even people with the same medical condition may report different QoL due to different expectations (Carr et al., 2001). An individual’s expectations strongly influence the evaluation of and satisfaction with their current situation. Therefore, the Calman Gap may be explained either by worsening of the current situation due to changes in health or personal life, or by failing to analogously adjust the desired situation. Adaptation of the desired situation can be beneficial in the face of progressive or chronic illness, where the previously desired state can no longer be reached and expectations must be adjusted accordingly (Brandtstädter and Rothermund, 1994; Carr et al., 2001; Etkind et al., 2019). Successful adaptation and adjustment of expectation can explain why people report high QoL even in the face of chronic illness and physical disability (Molton and Yorkston, 2016; Puvill et al., 2016). Thus, the Calman Gap is a valuable measure of QoL because it describes the relationship between experience and expectation and the factors that influence both.

Although QoL is a highly individual construct, certain factors have been identified that influence it across a multitude of patients, with several studies highlighting the importance of mental health components in particular (Schrag et al., 2000; Martinez-Martin et al., 2011; Puvill et al., 2016). Depressive symptoms in particular have been linked to worsening of QoL, eclipsing the impact of physical health (Kuopio et al., 2000; Puvill et al., 2016). In a previous study, we confirmed the influence of depression on the Calman Gap in people with Parkinson’s disease and epilepsy as well as healthy controls (Prell et al., 2020). With regards to the Calman Gap, depression has been mainly found to influence the perception of the current but not the desired situation, thus leading to an increased gap (Moore et al., 2005; Prell et al., 2020).

In the present study, we aimed to determine the influence of other parameters, in addition to depression, on the Calman gap, including resilience and health locus of control (HLC).

Resilience is a multi-faceted construct describing the ability to adapt to and cope with adverse events or severe stress. Resilience is associated with an increase in wellbeing and contributes to QoL by providing coping strategies and helping with adjustment (Ovaska-Stafford et al., 2021). Resilience may foster acceptance of a changes in current status and expectations (Windle et al., 2008), especially during chronic illness (Shamaskin-Garroway et al., 2016). Thus, resilience may influence the Calman Gap regarding both current and desired situation.

Another factor shaping health related expectations is the belief how much an individual is able to influence the course of their disease. HLC can be split into internal or adaptive HLC describing people who attribute successful solutions internally, and external or maladaptive HLC with patients showing the opposite pattern of attribution (Rotter, 1966; Muldoon et al., 2005; Groth et al., 2019). HLC describes the belief that a person can influence their health and is associated with better mental health status (Groth et al., 2019). HLC is intricately linked to self-efficacy; persons who perceive a situation as controllable tend to have different expectations regarding outcomes (Schmidt et al., 2014; Groth et al., 2019).

Thus, to understand what shapes the perception of current and desired states to form the Calman Gap, we analyzed the influence of sociodemographic factors, depression, resilience and HLC on the Calman Gap for a cohort of older patients with neurological disorders. Based on the current literature and our previous study, we expected depression to increase the gap by lowering current perception, and we expected resilience and internal HLC to counteract the effects of depression and decrease the gap by lowering the desired perception.

## Materials and Methods

### Study Design and Participants

This observational study was approved by the ethics committee of the Jena University Hospital (approval number 5290-10/17) and conducted according to the Declaration of Helsinki. Patients enrolled in the NeuGerAdh study (*n* = 910) (Prell, 2019; Prell and Schönenberg, 2022) were screened for eligibility and 100 participants were randomly selected. Briefly, the observational longitudinal NeuGerAdh study explores predictors of medication non-adherence in hospitalized older adults with neurological disorders. For this purpose the patients received a comprehensive geriatric assessment during hospital stay (baseline assessment) and follow-up telephone interviews after discharge. For our analyzed subsample, the inclusion criteria were age 60 years or older and no or only mild cognitive impairment as indicated by Montreal cognitive assessment, MoCA, >21 points (Nasreddine et al., 2005). Participants were contacted *via* telephone between November and December 2020. A face-to-face interview was not possible due to the COVID-19 pandemic restrictions. Six participants were excluded due to unwillingness or inability to provide valid data *via* telephone, leading to 94 analyzed datasets.

### Questionnaires

The following parameters were derived from the baseline assessment: age, gender, marital status (single, divorced/widowed, or married), level of education (high: German *Abitur* or university; medium: German *Realschule* or General Certificate of Secondary Education; low: German *Hauptschule* or no school) and employment status, health-related QoL assessed by the Short Form-36 (SF-36) (Ware and Sherbourne, 1992) and the MoCA to assess cognition (Nasreddine et al., 2005).

During the telephone interview, questionnaires for resilience, HLC and depression were administered in addition to questions regarding the Calman gap (see below). The German Fragebogen zur Erfassung gesundheitsbezogener Kontrolle (FEGK) was used to determine HLC. It includes 10 statements which are scored on a six-point Likert scale from 1 = “very true” to 6 = “very false.” The FEGK assess HLC on two subdomains, internal and external HLC. High sum values indicate high HLC and low sum values indicate low HLC. Internality refers to the belief in self-control of health-related events. Externality relates to the perception that health mainly depends on the actions of other persons, e.g., physicians, fate or chance (Ferring, 2003).

Resilience was assessed with the German Resilience Scale 11 (RS11), a questionnaire comprised of 11 statements scored on a 7-point Likert Scale ranging from 1 = “no, I do not agree at all” to 7 = “yes, I agree completely.” For the RS11, a sum score is calculated, with higher values indicating higher levels of resilience (von Eisenhart Rothe et al., 2013).

The PHQ-9 questionnaire was used to detect current depression levels. Its nine items cover the DSM-IV criteria for the diagnosis of depression. The cumulative score can range from 0 to 27 and assigns patients to the categories “no depression” (<10), “mild depression” (10–19) and “severe depression” (20–27) (Gräfe et al., 2004; Kroenke, 2012).

### Assessment of Calman Gap

Calman gaps were determined for four health-related domains [Fitness, General Health, Pain, and restrictions in Activities of Daily Living (ADL)], as well as satisfaction with Finances, Leisure activities, and relationship with Family (Prell et al., 2020). For every item, the participants were asked to rate both the current and the desired state on a scale from 0 to 100, with higher numbers indicating higher satisfaction (see Supplementary Table 1). The items were generated on the basis of other validated QoL questionnaires, such as the Fragebogen zur Lebenszufriedenheit (Life Satisfaction Questionnaire) (Fahrenberg et al., 2000), the Befragung “Generation 50 plus: Lebensqualität und Zukunftsplanung in Düsseldorf” (Generation 50 Plus: QoL and Future Planning in Düsseldorf) (Gloschinski, 2011); the SF-36 (Ware and Sherbourne, 1992) and the Calman gap questionnaire used in our previous study (Prell et al., 2020). The items were cross-validated with the SF-36 domains.

### Statistical Analyses

SPSS (version 23.0; IBM Corporation, Armonk, NY, United States) and R 3.6.2 (R Foundation for Statistical Computing, Vienna, Austria) were used for statistical analyses. Descriptive statistics were used to describe the patient cohort. Continuous values are expressed as mean and standard deviation (SD), categorical variables are presented as numbers and percentages. Patients were categorized into two HLC groups: internal and external HLC. Conformity with assumptions for regression models was assessed with the R-package *performance*; while no relevant outliers were detected using Cook’s Distance, high levels of multicollinearity based on the Variance Inflation Factor (VIF) were found in all models (see Supplementary Figure 1; Lüdecke et al., 2021).

Confirmatory factor analysis was calculated using the Lavaan package in R Version: 0.6-7. To evaluate model fit, several fit indices were considered: the χ^2^-likelihood ratio statistic, the comparative fit index (CFI), the Tucker-Lewis index (TLI), the root mean square error of approximation (RMSEA) and the standardized root mean square residual (SRMR) (Hu and Bentler, 1999). Good model fit was indicated for a non-significant χ^2^ test, an RMSEA less than 0.05, CFI/TLI above 0.90 to 0.95, and SRMR less than 0.08 (Rosseel, 2012).

Elastic net models were calculated to assess factors associated with current perception, desired perception, and the health-related gap as dependent variables and the following independent variables: age, gender, education, MoCA, HLC internal, HLC external, Resilience Sum, PHQ-9 Sum, and current and desired perception of the respective variable. Elastic net regularization has the advantage of parsimonious models that are easier to interpret. It is suitable for models with a large number of predictors even in small sample sizes, as it prevents overfitting and selects variables by shrinking parameters toward zero. Ten-fold cross validation was used to select the most appropriate model with the lowest mean cross-validated error. Variables remain in the model if the prediction error decreases over the cross-validation samples. Another advantage of the elastic net algorithm is that, unlike LASSO or least square regressions, it handles correlated variables well by either including all of them with comparable regression coefficients or excluding them all from the model. For this purpose, the penalty parameters Lambda and Alpha were not set in advance but were determined in an iterative process in which all possible combinations were tested until the best combination was found (Zou and Hastie, 2005). Regressions coefficients of the model with 95% confidence intervals (CI) were reported. Elastic net regularization was performed using the *glmnet* package in R 3.6.2 (Friedman et al., 2010; Wickham, 2016; Lüdecke et al., 2021).

## Results

### Cohort Description

The clinical and demographic characteristics of the cohort are shown in Table 1. Most patients were male, married, retired, lived together with a partner, and had middle or high education levels. According to the PHQ-9, 87 (92.6%) persons reported no or mild depression, 6 (6.4%) reported moderate, and 1 (1.1%) severe depression. The majority (*n* = 79, 84%) of study participants had internal HLC and only 15 (16%) had external HLC. According to the medical records, the main neurological diagnoses were cerebrovascular disorders (37, 39.4%), movement disorders (28, 29.8%), peripheral or neuromuscular disorders (18, 19.1%), and miscellaneous diagnoses (10, 10.6%).

**TABLE 1:** Clinical and demographic characteristics (*N* = 94).

		*n*	(%)		
Sex	Female	36	38.3%		
	Male	58	61.7%		
Marital status	Single	4	4.3%		
	Married	66	70.2%		
	With other partner	3	3.2%		
	Divorced, widowed	21	22.3%		
Living situation	Alone	18	19.6%		
	With partner	73	79.3%		
	Others	1	1.1%		
Nearby relatives	Yes	74	78.7%		
	No	20	21.3%		
Education	Low	23	24.5%		
	Medium	33	35.1%		
	High	38	40.4%		
Employment	Full time	7	7.4%		
	Partly employed	0	0.0%		
	Not employed	3	3.2%		
	Pensioned	84	89.4%		

		**M**	**SD**	**Maximum**	**Minimum**

Age (years)		71.8	7.7	91.0	60.0
HLC internal		4.6	0.5	5.8	3.2
HLC external		3.4	0.9	5.4	1.0
Resilience		62.8	8.8	79.0	33.0
PHQ		3.6	3.7	15.0	0
MoCA		24.58	2.17	29	21

		**Current state**		**Desired state**	
**QoL domain**		**M**	**SD**	**M**	**SD**

Fitness		57.39	19.51	77.66	15.96
Health		56.65	21.97	78.40	16.74
Pain		61.49	16.74	90.11	18.29
ADL		60.11	28.45	81.60	22.59
Finances		73.70	22.25	82.97	17.87
Leisure		72.45	25.93	86.91	14.81
Family		87.66	19.81	95.00	14.12

*HLC, health locus of control; PHQ, patient health questionnaire; MoCA, montreal cognitive assessment; QoL, quality of life; ADL, activities of daily living.*

### Compound Health-Related Gap

**FIGURE 1:**
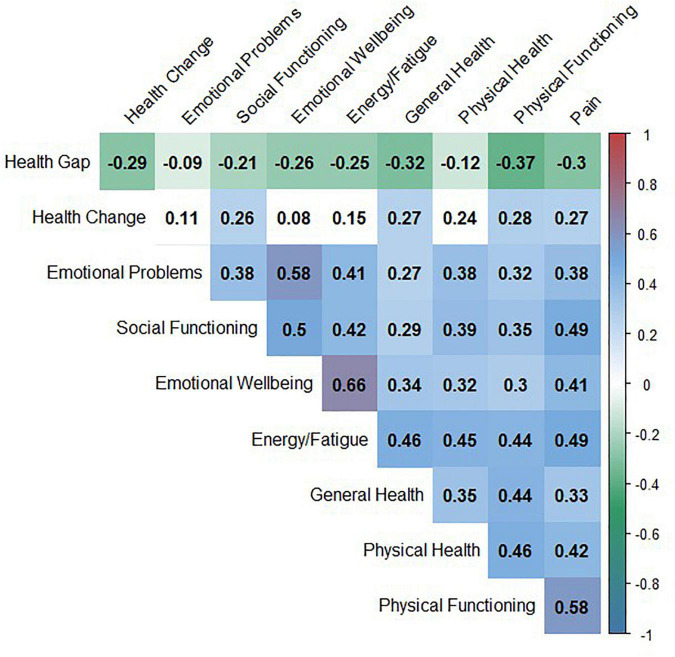
Correlation matrix: univariate correlation between the compound Health Gap and SF-36 domains.

A confirmatory factor analysis revealed that the four items Fitness, General Health, ADL and, to a lesser extent, Pain load on one factor (Cronbach’s alpha = 0.73). A second model with the three items Fitness, General Health, and ADL (without item Pain) showed better model fit, with a higher Cronbach’s alpha of 0.82 (Table 2). Therefore, the means of Fitness, General Health, and ADL values were used to build a compound health-related gap. This compound health-related gap correlated with other measures of health-related QoL (SF-36 domains) (Figure 1). The pain gap correlated with the SF-36 pain domain (*r* = −0.32, *p* = 0.001).

**TABLE 2:** Fit indices for models tested *via* confirmatory factor analysis.

Fit index	A: one factor, 4 items*		B: one factor, 3 items*	
χ^2^	0.627		n.a.	
df	2		3	
CFI	1.000		1.000	
TLI	1.021		1.000	
RMSEA	<0.001		<0.001	
SRMR	0.016		<0.001	
AIC	3244.978		2330.411	
**Standardized factor loadings**				
Fitness	0.840	*p* < 0.001	0.844	*p* < 0.001
General health	0.807	*p* < 0.001	0.811	*p* < 0.001
Pain	0.341	*p* < 0.001	Not entered	
ADL	0.872	*p* = 0.001	0.865	*p* < 0.001

*df, degrees of freedom; CFI, comparative fit index; TLI, tucker-lewis index; RMSEA, root mean square error of approximation; SRMR, standardized root mean square residual; AIC, akaike’s information criterion.*

*Items: Fitness, General Health, ADL, activities of daily living, (Pain).

### Prediction of Current and Desired States

To explore factors contributing to the patients’ rating of current and desired states, regression analyses were calculated *via* elastic net regularization for each of the seven domains. For all domains, the current and desired state were significantly linked (*p* < 0.01 for all domains, see also Supplementary Figure 2). After variable selection *via* elastic net regularization, depression as measured by the PHQ remained in the model for the current perception of the domains Fitness (*p* = 0.18), pain (*p* = 0.07), ADL (*p* = 0.43), and Family (*p* = 0.06). Of the sociodemographic factors, age remained in the model for the domains Fitness (*p* < 0.01), Health (*p* < 0.01), Pain (*p* = 0.22), ADL (*p* = 0.01), and Family (*p* = 0.19). Gender remained included in the models for Pain (*p* = 0.41), ADL (*p* = 0.61), and Family (*p* = 0.07), whereas living with a partner was part of the models for Fitness (*p* = 0.83), Pain (*p* = 0.05), ADL (*p* = 0.49), and Family (*p* = 0.61). Education was relevant in the models for Fitness (*p* = 0.16), Health (*p* = 0.16), Pain (0.02), and ADL (*p* = 0.05).

Health locus of control remained in the models for Fitness (internal HLC *p* = 0.16, external HLC *p* = 0.26), Health (internal HLC *p* = 0.09, external HLC *p* = 0.06), Pain (internal HLC *p* = 0.03), ADL (internal HLC *p* = 0.26, external HLC *p* = 0.23), and Finances (external HLC *p* = 0.12).

Resilience further remained influential in the current perception of Fitness (*p* = 0.37) and Family (*p* = 0.02).

For desired state, in addition to current perception (*p* < 0.01 for all domains), living with a partner influenced desired Health (*p* = 0.12), depression influenced Pain (*p* = 0.05), Finances (*p* = 0.08) and Family (*p* = 0.15), while HLC remained in the model for ADL (internal HLC *p* = 0.09) and Family (internal HLC *p* = 0.55). Additionally, age (*p* = 0.47), gender (*p* = 0.17), education (middle = 0.42 and low = 0.50), resilience (*p* = 0.01) and cognition (MoCa, *p* = 0.41) remained in the model for desired Family state.

**FIGURE 2:**
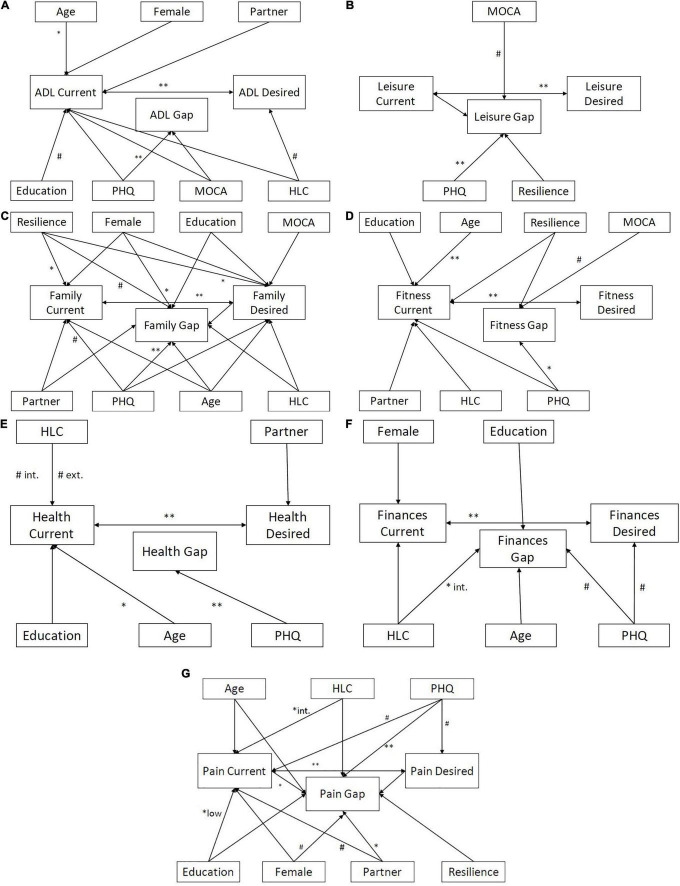
Detailed associations between the current state, desired state, and Calman gap. **(A)** ADL gap. **(B)** Leisure gap. **(C)** Family gap. **(D)** Fitness gap. **(E)** Overall health gap. **(F)** Finances gap. **(G)** Pain gap. ^#^*p* < 0.1, *p < 0.05, and **p < 0.01 based on Elastic Net models for current, desired and gap; ADL, activities of daily living (int = internal, ext = external); HLC, Health Locus of Control; MOCA, Montreal Cognitive Assessment; PHQ, Patient Health Questionnaire.

Detailed results of regression analyses are given in the Supplementary Tables 2, 3 and in Figure 2.

Regarding the health-related domains, the perception of current Fitness (Figure 2D) was positively influenced by age and desired Fitness state; to a lesser extent resilience, depression, HLC, living situation and education played a role for the rating of the current Fitness state. Similarly, perception of current General Health (Figure 2E) improved with increasing age and increasing desired General health, with both external and internal HLC playing a modulating role. In terms of ADL state (Figure 2A), mainly higher age and higher desired ADL state were associated with perception of a better current ADL state. The current Pain state was rated worse with higher expectations toward improvement of Pain (higher desired state), lower education level and less internal HLC (Supplementary Table 2).

The desired Fitness was determined by current Fitness. Desired General Health was associated with current health and living situation; living with a partner was associated with decreased expectations of desired General Health. Desired ADL was positively associated with internal HLC and current ADL state. Desired Pain state was associated with depression and current Pain state (Supplementary Table 2).

### Prediction of the Calman Gaps

**FIGURE 3:**
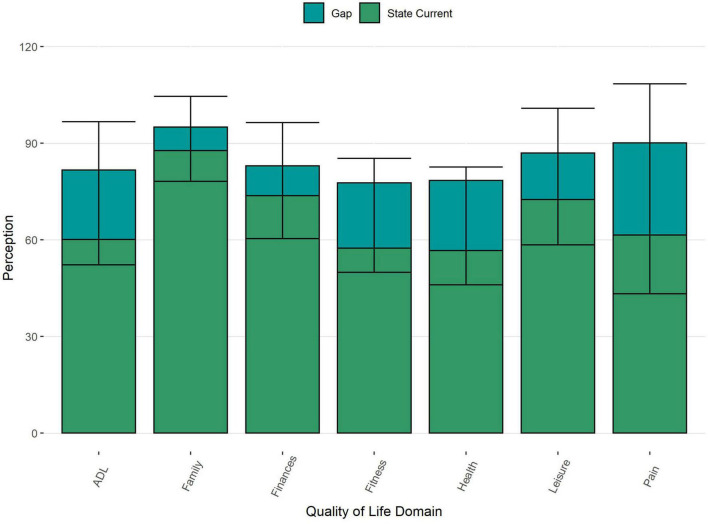
Distribution and mean current and desired Quality of Life and the mean gap (with SD) for each domain. ADL, activities of daily living; the height of each bar represents the mean desired state.

A gap between current and desired state was reported for all obtained QoL domains (Figure 3). The largest gaps were observed for Pain and General Health and the smallest gap was present for Family.

To understand the nature of these gaps, separate regression analyses *via* elastic net regularization were calculated for each gap (see Supplementary Table 3 and Figure 2) and the combined health-related gap (Table 3). All gaps were increased by depression. In addition, the Finance gap (Figure 2F) was associated with internal HLC, the Family gap (Figure 2C) was reduced in female patients, and the Pain gap (Figure 2G) was associated with current pain perception and living together with a partner. Cognition, sociodemographic factors, HLC and resilience further influenced the Fitness (Figure 2D), Pain, Finances, Leisure (Figure 2B) and Family gaps. The influencing factors for the health-related domains are summarized in Supplementary Figure 3.

**TABLE 3:** Predictors of health-related Calman gap.

	*Est*	Lower 95% CI	Upper 95% CI	*t*-value	*p*
Intercept	5.67	–40.2	51.86	0.24	0.81
Resilience	–0.18	–0.80	0.21	–0.92	0.36
PHQ	1.73	0.71	2.75	3.33	<0.01
ADL current	–0.12	–0.23	0.00	–1.97	0.05
MoCA	1.12	–0.44	2.69	1.41	0.16

*PHQ, patient health questionnaire; ADL, activities of daily living; MoCA, montreal cognitive assessment.*

*Entered independent variables: age, gender, living situation, education, resilience, health locus control internal and external, PHQ, MOCA, current and desired ADL, fitness, and health.*

*χ^2^(4) = 6459.40, p = 0.00, Pseudo-R^2^ (Cragg-Uhler) = 0.25, Pseudo-R^2^ (McFadden) = 0.03, AIC = 745.05, BIC 759.99.*

In the elastic net model [χ^2^(4) = 6459.40, *p* < 0.001, Pseudo-*R*^2^ (Cragg-Uhler) = 0.25, Pseudo-*R*^2^ (McFadden) = 0.03, AIC = 745.05, BIC 759.99], depression was associated with a higher compound health-related Calman gap (ß = 1.73, *p* < 0.001, CI [0.71; 2.75]). After entering the current and desired states into the model, a higher compound health-related Calman gap was associated with depression, problems in ADL, higher MoCa, and lower resilience (Table 3).

A one-way MANOVA showed a statistically significant effect of the health-related Calman gap on the combined dependent variable of the other non-health gaps (Family, Leisure, Finance), [*F*_(3, 90)_ = 14.63, *p* < 0.001, partial η^2^ = 0.328, Wilk’s Λ = 0.672]. In particular, a significant association was observed between Health-gap and Leisure gap (*p* < 0.001, partial η^2^ = 0.29), Health-gap and Family (*p* = 0.009, partial η^2^ = 0.072), but not between Health-gap and Finance gap (*p* = 0.052). After entering age (*p* = 0.49), gender (*p* = 0.032, partial η^2^ = 0.098), living situation (*p* = 0.193), and PHQ-9 (*p* < 0.001, partial η^2^ = 0.198) into the model, only the association between Health-gap and Leisure activities remained significant (*p* < 0.001, partial η^2^ = 0.173).

## Discussion

The aim of the present study was to assess the influence of depressive mood, resilience, HLC, and sociodemographic factors on the Calman Gap in elderly patients with neurological disorders. For this purpose, the impact of these parameters on seven specific gaps and a compound health-related gap was analyzed. The items of this compound health gap correlated with the SF-36 domains, indicating that it captured health-related QoL aspects (Ware and Sherbourne, 1992).

A gap, meaning a discrepancy between current and desired status, was present for all studied domains, indicating that the participants were not fully satisfied with their current situation. This finding is in line with other studies showing a potentially diminishing impact of age and chronic illness on QoL (Westendorp et al., 2013; Steptoe et al., 2015). However, it is of note to say that on average, patients did not report the highest possible levels regarding the desired state, indicating a lowering of expectations and adjustment of evaluation (Heckhausen et al., 2010; Steptoe et al., 2015; Wettstein et al., 2019). To understand the exact mechanisms behind this discrepancy between the current and desired states, we analyzed multiple factors and their influence on this gap.

As revealed in previous studies (Moore et al., 2005; Prell et al., 2020), we found depression to be the main driving force of the Calman gaps. Depression is known to be one of the key factors influencing life satisfaction in advanced age, more so than physical health (Kuopio et al., 2000; Martinez-Martin et al., 2011; Puvill et al., 2016). In line with our previous study (Prell et al., 2020), the present results indicate that depression is closely related to the Calman gap and the rating of the current state. In addition, the perception of the current state was linked to the desired state, with higher desired states leading to a better evaluation of the current state (Voss et al., 2017). Similarly, current perception positively influenced desired perception. Thus, a desired state which lies above the current state but has been adjusted to not lie entirely out of reach may motivate patients (Heckhausen et al., 2010; Kornadt et al., 2015). As a gap was still present for all domains, it is important to keep in mind that in this particular cohort of ill patients (formerly hospitalized), a discrepancy between current and desired state is to be expected as the current health is reduced compared to the healthy population (Ellert and Kurth, 2013). Thus, as a mechanism for coping with changes due to advancing age and declining health, it is important to build expectations that lie within realistic reach in this cohort with health problems (Heckhausen et al., 2010; Kornadt et al., 2015). The interpretation of this delicate balance, i.e., the interpretation of the gaps themselves, may be linked to depression. The subordinate influence of depression on the current and desired states in comparison to other studies (Moore et al., 2005; Prell et al., 2020) may be explained by the low levels of depression reported in this particular cohort; patients were not severely depressed and also did not report lowest level of satisfaction with current state. It is possible that the influence of depression may overpower the motivational aspect of expectations at a certain stage and thus lead to the previously reported different perception of the current state.

Furthermore, increasing age was found to be a protective factor positively influencing the perception of the current state of fitness, health and ADL. This finding reflects the idea that younger people have higher expectations regarding their lifestyle and health, as they are not yet at an age where they expect age-related declines in health. Thus, younger patients with declining health may either interpret their current situation as worse or fail to adjust their desires accordingly. In contrast, older adults are known to be more resilient as they learn how to deal with losses and setbacks because they are both more expected and more frequent (Steptoe et al., 2015; Wettstein et al., 2019; Ovaska-Stafford et al., 2021). Therefore, older adults have adjusted their perception to the decline in health and shifted their goals and comparison group (Brandtstädter and Rothermund, 1994). Similarly, the presence of a partner led to higher perception of current pain in the present study, as having someone potentially healthier by their side may lead patients to maladaptively compare their health state to someone else. This idea is also reflected in a nationwide German survey on QoL indicating that QoL ratings were lower for middle aged participants than for older persons (Ellert and Kurth, 2013).

Contrary to our expectations, resilience was most strongly related to the social domain of family. Resilience mainly influenced the Family domain through a better perception of current Family status. However, resilience also reduced the desired status, indicating that patients with high levels of resilience were less dependent on social support. Similarly, higher levels of resilience may decrease the leisure gap, showing that in this cohort, resilience was more related to social constructs than health. This aspect of external resilience is in line with other studies reporting psychosocial aspects of resilience but with mixed results regarding the impact on physical functioning (Battalio et al., 2017; Ovaska-Stafford et al., 2021). Thus, resilience may serve as a protective factor, as shown by the influence of resilience on fitness and pain in the current study (Steptoe et al., 2015; Molton and Yorkston, 2016; Ovaska-Stafford et al., 2021). However, in this cohort of elderly persons with neurological disorders, it is possible that other factors such a depression or physical health overpower the influence of resilience on health factors (Terrill et al., 2016).

The influence of HLC differed between variables. External HLC has previously been described as maladaptive as it may prevent appropriate coping and formation of realistic expectations (Groth et al., 2019). In contrast, external HLC and belief in the influence of external factors may be beneficial in chronic illness, where trust in external factors such as doctors and medication is important. Additionally, parameters of control and self-efficacy differ between younger and older persons (French et al., 2014). As shown in our study, external HLC positively influenced the perception of current health, whereas internal HLC reduced current health state. In advanced age and illness, internal HLC may put pressure on a person to change something that is essentially out of their power and thus lead to lower levels of current health perception (Leventhal et al., 1998; Richardson et al., 2017). In contrast, internal HLC may positively influence desired ADL, because that patients feel more self-efficacy when it comes to activities rather than actual health status. Likewise, research suggests that internal HLC may not be able to mediate health-related behavior, instead proposing that the value of health serves as a stronger motivator for health-related behavior or interpretations. Furthermore, it is important to keep in mind the potential discrepancy between a general belief in control and a situation-specific control (Wallston and Wallston, 1982). The questionnaire used mainly asked about the general perception of health-related control. It is therefore possible that participants generally believe they have control over their health, but this overall belief may not translate into the actual perception of their specific, current health situation (Wallston, 1992; Ferring, 2003). HLC is a multidimensional construct (Otto et al., 2011) and belief in external HLC factors can be further split into change/fate or belief in other persons, such as doctors (Náfrádi et al., 2017). Thus, it is necessary for future studies to disentangle the attribution of external HLC to truly understand the influence it has on health perception and behavior.

Finally, we combined distinct domains to build a compound health-related Calman gap. Interestingly, fit indices (as shown in Table 2) which are less sensitive to the number of estimated parameters, revealed that the three-item model of the compound health-related Calman gap (excluding Pain) fit better than a four-item model including Pain. This finding indicates that pain should be treated as an individual domain, separate from other health-related factors such as ADL, General Health and Fitness (DeVine et al., 2011; Müller et al., 2017). As suggested by other studies, pain is an individual interpretation rather than an objective physical function or health state and is linked to mood and depression. As pain may also interfere with participation in daily life, it worsens overall QoL (Bombardier et al., 2010; Turk et al., 2016; Karayannis et al., 2017; Farrar, 2019). In a similar manner, in our study pain showed the largest Calman gap and the second highest average desired state after Family, indicating that patients were less willing to compromise on pain levels and desired to have no pain at all if possible. In line with the relationship between pain and mood (Bombardier et al., 2010; Turk et al., 2016), assessment of current pain was mainly related to the desired pain state and depression, and pain perception further increased with internal HLC and living with a partner.

### Limitations

This study has several limitations. The cross-sectional design allows an insight into the patients’ current perception, but does not allow for any interpretations of causality or longevity. Furthermore, while QoL and adjustment of expectations are highly relevant in the cohort of older adults with neurological disorders, the selection of a specific cohort reduces the generalizability of the effects. While the effect of depression on the Calman Gaps was prominent, the vast majority of patients only showed little to very mild depression according to the PHQ-9, thus the influence of more severe depression levels on QoL and the according Calman Gap remains unclear.

## Conclusion

Overall, the compound health-related Calman gap was mainly influenced by depression and ADL, indicating that the interpretation of health and its impact on QoL is strongly related to mood and the ability to participate and perform daily activities (Schrag et al., 2000; Müller et al., 2017). As shown in this study, the presence of a Health gap extends its influence into other non-health domains as it restricts participation (Müller et al., 2017), especially regarding leisure activity and family. This finding highlights the importance of health perception as it is not restricted to health itself but spreads across many aspects of life. Attitudes toward aging and its corresponding expectations have been shown to influence cognition, psychological and physical health factors, even on a long-term scale (Robertson et al., 2016), suggesting that the perception of aging and the expectations that accompany it play a pivotal role for healthy aging. Perceived QoL is linked to expectations and motivational aspects derived from their proper adjustment, it is important to support patients in finding an optimal balance between appropriate goal adjustment on the one hand, and motivational aims on the other hand. Several sociodemographic factors as well as perception of control and resilience further influence this health perception. Of note, as the interpretation of this gap may be mediated by depression, it is crucial to stabilize patients’ mood and counteract depression already at a low level to facilitate appropriate coping. These results are highly relevant for intervention studies aiming to improve QoL in persons with chronic illness, as they suggest that a delicate balance between adjustment of expectations according to the respective health state, and an upkeep of motivation and potentiality must be found. Furthermore, depression may disturb this delicate balance and should thus be treated appropriately.

## Data Availability Statement

The data collected for this particular analysis are freely available for scientific use at the following link: https://osf.io/e6g3n/ (Schönenberg and Prell, 2022).

## Ethics Statement

The studies involving human participants were reviewed and approved by the Ethics Committee of Jena University Hospital, Jena, Germany. The patients/participants provided their written informed consent to participate in this study.

## Author Contributions

TP, AS, and UT: design of the study. AS, UT, and HZ: data collection. AS and TP: analysis. AS: writing – original draft. TP and HZ: writing: review and editing. All authors have read and agreed to the final version of the manuscript.

## Conflict of Interest

The authors declare that the research was conducted in the absence of any commercial or financial relationships that could be construed as a potential conflict of interest.

## Publisher’s Note

All claims expressed in this article are solely those of the authors and do not necessarily represent those of their affiliated organizations, or those of the publisher, the editors and the reviewers. Any product that may be evaluated in this article, or claim that may be made by its manufacturer, is not guaranteed or endorsed by the publisher.
