# Supplementary material for: Impact of Depression, Resilience, and Locus of Control on Adjustment of Health-Related Expectations in Aging Individuals With Chronic Illness
Source: Front Psychol. 2022 Apr 28;13:867785. doi: 10.3389/fpsyg.2022.867785 (PMC9097897; doi:10.3389/fpsyg.2022.867785)
Supplement: Supplementary file 1 [file Data_Sheet_1.docx]

Supplementary Material

**Supplement Table 1.** Translation of Gap Domain Items.

1. Fitness: how fit do you currently feel? 0 = I cannot stand up without help, 100 = I could run a marathon tomorrow
2. General Health: how would you rate your general health? 0 = extremely bad 100 = extremely good
3. Pain: are you in pain? 0 = never 100 = daily*
4. Activities of Daily Living: does your health restrict you in your daily activities? 0 = I am completely restricted 100 = I am not restricted at all
5. Finances: are you content with your financial situation? 0 = No, I cannot afford anything 100 = Yes, I can afford anything I want
6. Leisure: how satisfied are you with your leisure activities? 0 = extremely dissatisfied 100 = extremely satisfied
7. Family: how content are you with your relationship with your family? 0 = extremely dissatisfied 100 = extremely satisfied

*Item was inversed later

Note: each item was rated for both the current situation and the desired situation on a scale from 0 to 100.

**Supplement Table 2a**. Predictors of Current state.

| **Variable** |  | **Est.** | **95% CI** | **t** | ***p*** |
| --- | --- | --- | --- | --- | --- |
| Fitness | Intercept | -42.48 | -101.59; 16.62 | -1.41 | .16 |
|  | Age | .68 | .24; 1.12 | 3.03 | **< .01** |
|  | Living with Partner | -.94 | -9.47; 7.59 | -.22 | .83 |
|  | Education Middle | 5.02 | -1.91; 11.95 | 1.42 | .16 |
|  | Resilience | .19 | -.23; .61 | .89 | .37 |
|  | HLC Int. | -4.54 | -10.82; 1.73 | -1.42 | .16 |
|  | HLC Ext. | 2.10 | -1.52; 5.73 | 1.14 | .26 |
|  | PHQ | .70 | -.31; 1.72 | 1.36 | .18 |
|  | Fitness Desired | .64 | .43; .85 | 6.04 | **< .01** |
|  | X²(8) = 13860.96, p < .001, Pseudo-R² (Cragg-Uhler) = .39, Pseudo-R² (McFadden) = .06,  AIC = 797.53, BIC = 822.96 | | | | |
| Health | Intercept | -38.89 | -85.96; 8.19 | -1.62 | .11 |
|  | Age | .69 | .24; 1.14 | 3.01 | **< .01** |
|  | Education Middle | 5.20 | -1.99; 12.39 | 1.42 | .16 |
|  | HLC Int. | -5.65 | -12.01; .72 | -1.74 | .09 |
|  | HLC Ext. | 3.63 | -.16; 7.41 | 1.88 | .06 |
|  | Health Desired | .74 | .53; .94 | 7.05 | **< .01** |
|  | X²(5) = 20277.19, p < .001, Pseudo-R² (Cragg-Uhler) = .45, Pseudo-R² (McFadden) = .07, AIC = 804.05, BIC = 821.85 | | | | |
| Pain | Intercept | 106.37 | 9.79; 203.04 | 2.16 | .03 |
|  | Age | -.56 | -1.44; 0.32 | -1.24 | .22 |
|  | Female | -5.66 | -19.03; 7.71 | -.83 | .41 |
|  | Living With Partner | -16.53 | -32.92; -.14 | -1.98 | .05 |
|  | Education Middle | 8.74 | -.634, 23.83 | 1.14 | .26 |
|  | Education Low | 20.84 | 3.20, 38.48 | 2.32 | **.02** |
|  | HLC Int. | -14.23 | -26.63; -1.83 | -2.25 | **.03** |
|  | PHQ | -1.71 | -3.57; .14 | -1.81 | .07 |
|  | Pain Desired | .83 | .47; 1.18 | 4.53 | **< .01** |
|  | X²(8) = 37715.86, p < .001, Pseudo-R² (Cragg-Uhler) = .32, Pseudo-R² (McFadden) = .04, AIC = 922.65, BIC = 948.08 | | | | |
| ADL | Intercept | -66.04 | -161.98; 29.89 | -1.35 | .18 |
|  | Age | .86 | .25; 1.47 | 2.75 | **.01** |
|  | Female | 2.42 | 6.86; 11.69 | .51 | .61 |
|  | Living with Partner | -4.00 | -15.34; 7.34 | -.69 | .49 |
|  | Education Middle | 9.73 | .31; 19.16 | 2.02 | .05 |
|  | MoCA | .37 | -1.83; 2.58 | .33 | .74 |
|  | HLC Int. | -5.01 | -13.70; 3.67 | -1.13 | .26 |
|  | HLC Ext. | 3.12 | -1.97; 8.21 | 1.20 | .23 |
|  | PHQ | .54 | -.80; 1.88 | .79 | .43 |
|  | ADL Desired | .37 | .57; .99 | 7.33 | **< .01** |
|  | X²(9) = 32558.70, p < .001, Pseudo-R² (Cragg-Uhler) = .48, Pseudo-R² (McFadden) = .07, AIC 805.28, BIC = 832.65 | | | | |
| Finances | Intercept | -.76 | -22.29; 20.76 | -.07 | .94 |
|  | Female | 5.53 | -1.8; 12.90 | 1.47 | .15 |
|  | HLC Ext. | 3.14 | -.77; 7.05 | 1.57 | .12 |
|  | Finances Desired | .74 | .54; .95 | 7.26 | **< .01** |
|  | X²(3) = 18900.20, p < .001, Pseudo-R² (Cragg-Uhler) = .41, Pseudo-R² (McFadden) = .06, AIC = 809.37, BIC = 822.08 | | | | |
| Leisure | Intercept | -15.34 | -41.10; 10.43 | -1.17 | .25 |
|  | Leisure Desired | 1.01 | .72; 1.30 | 6.78 | **< .01** |
|  | X²() = 20815.87, p < .001, Pseudo-R² (Cragg-Uhler) = .33, Pseudo-R² (McFadden) = .04, AIC 845.73, BIC = 853.36 | | | | |
| Family | Intercept | -28.67 | .70.13; 12.79 | -1.36 | .18 |
|  | Age | -.23 | -.57; .11 | -1.33 | .19 |
|  | Female | 4.98 | -.38; 10.34 | 1.82 | .07 |
|  | Living With Partner | -1.74 | -8.41; 4.94 | -.51 | .61 |
|  | Resilience | .40 | .07, .73 | 2.37 | **.02** |
|  | PHQ | .77 | -.01; 1.56 | 1.92 | .06 |
|  | Family Desired | 1.10 | .91; 1.28 | 11.63 | **< .01** |
|  | X²(6) = 22708.67, p < .001, Pseudo-R² (Cragg-Uhler) = .62, Pseudo-R² (McFadden) = .11, AIC 751.58, BIC = 771.92 | | | | |
| Note: HLC = Health Locus of Control, PHQ = Patient Health Questionnaire, MoCA = Montreal Cognitive Assessment,  ADL = Activities of Daily Living, CI = Confidence Interval | | | | | |

**Supplement Table 2b**. Predictors of Desired state.

| Variable |  | **Est.** | **95% CI** | **t** | ***p*** |
| --- | --- | --- | --- | --- | --- |
| Fitness | Intercept | 53.37 | 44.47; 62.04 | 12.07 | < .01 |
|  | Fitness Current | .42 | .28; .57 | 5.80 | < .01 |
|  | X²(1) = 6338.54, p < .001, Pseudo-R² (Cragg-Uhler) = .27, Pseudo-R² (McFadden) = .04, AIC = 763.24, BIC = 770.87 | | | | |
| Health | Intercept | -58.24 | 48.40; 68.08 | 11.60 | < .01 |
|  | Living With Partner | -5.44 | -12.20; 1.31 | -1.58 | .12 |
|  | Health Current | .43 | .31; .56 | 6.69 | **< .01** |
|  | X²(2) = 9327.08, p < .001, Pseudo-R² (Cragg-Uhler) = .36, Pseudo-R² (McFadden) = .05, AIC = 761.86, BIC = 772.03 | | | | |
| Pain | Intercept | 72.89 | 65.21; 80.56 | 18.61 | < .01 |
|  | PHQ | .90 | .01; 1.80 | 1.97 | .05 |
|  | Pain Current | .23 | .12; .32 | 4.74 | **< .01** |
|  | X²(2) = 6624.29, p < .001, Pseudo-R² (Cragg-Uhler) = .21, Pseudo-R² (McFadden) = .03, AIC = 797.60, BIC = 807.77 | | | | |
| ADL | Intercept | 23.86 | -7.68; 55.40 | 1.48 | .14 |
|  | HLC Int. | 5.77 | -.74; 12.28 | 1.74 | .09 |
|  | ADL Current | .52 | .39; .64 | 8.22 | **< .01** |
|  | X²(2) = 20561.47, p < .001, Pseudo-R² (Cragg-Uhler) = .43, Pseudo-R² (McFadden) = .06, AIC = 806.48, BIC = 816.65 | | | | |
| Finances | Intercept | 48.89 | 38.68; 59.10 | 9.39 | < .01 |
|  | PHQ | -.68 | -1.44; .08 | -1.76 | .08 |
|  | Finances Current | .50 | .37; .62 | 7.62 | **< .01** |
|  | X²(2) = 11960.39, p < .001, Pseudo-R² (Cragg-Uhler) = .40, Pseudo-R² (McFadden) = .06, AIC = 767.41, BIC = 777.59 | | | | |
| Leisure | Intercept | 63.04 | 55.71; 70.37 | 16.85 | < .01 |
|  | Leisure Current | .33 | .23; .42 | 6.78 | **< .01** |
|  | X²(1) = 6792.03, p < .001, Pseudo-R² (Cragg-Uhler) = .33, Pseudo-R² (McFadden) = .05, AIC = 740.46, BIC = 748.09 | | | | |
| Family | Intercept | 66.63 | 24.02; 109.24 | 3.06 | < .01 |
|  | Age | .12 | -.16; .39 | .83 | .41 |
|  | Female | -2.86 | -6.94; 1.22 | -1.38 | .17 |
|  | Education Middle | 1.89 | -2.68; 6.47 | .81 | .42 |
|  | Education Low | 1.89 | -3.62; 7.39 | .67 | .50 |
|  | Resilience | -.34 | -.58; -.10 | -2.76 | **.01** |
|  | HLC Int. | 1.14 | -2.63; 4.92 | .59 | .55 |
|  | PHQ | -.48 | -1.12; .016 | -1.46 | .15 |
|  | Family Current | .55 | .45, .65 | 10.99 | **< .01** |
|  | MoCA | -.41 | -1.37; .56 | -.82 | .41 |
|  | X²(9) = 11180.11, p < .001, Pseudo-R² (Cragg-Uhler) = .63, Pseudo-R² (McFadden) = .12, AIC = 685.39, BIC = 685.77 | | | | |
| Note: HLC = Health Locus of Control, PHQ = Patient Health Questionnaire, MoCA = Montreal Cognitive Assessment,  ADL = Activities of Daily Living, CI = Confidence Interval | | | | | |

**Supplement Table 3**. Predictors of the gaps for all domains.

| Variable |  | **Est.** | **95% CI** | **t** | **p** |
| --- | --- | --- | --- | --- | --- |
| Fitness | Intercept | 29.49 | 2.68; 50.30 | 2.16 | .03 |
|  | Resilience | -.31 | -.71; .10 | -1.47 | .14 |
|  | PHQ | 1.51 | .46; 2.55 | 2.82 | **.01** |
|  | MoCA | 1.42 | -.20; 3.03 | 1.72 | .09 |
|  | X²(3) = 6497.31, p < .001, Pseudo-R² (Cragg-Uhler) = .23, Pseudo-R² (McFadden) = .03, AIC = 749.77, BIC = 762.21 | | | | |
| Health | Intercept | 16.28 | 11.34; 21.22 | 6.46 | < .01 |
|  | PHQ | 1.51 | .56; 2.45 | 3.11 | **< .01** |
|  | X²(1) = 2950.25, p < .001, Pseudo-R² (Cragg-Uhler) = .10, Pseudo-R² (McFadden) = .01, AIC = 808.53, BIC = 816.16 | | | | |
| Pain | Intercept | -78.26 | -185.36; 28.84 | -1.43 | .16 |
|  | Age | -.23 | -1.06; .61 | - .53 | .60 |
|  | Female | 11.72 | - .84; 24.28 | 1.83 | .07 |
|  | Living With Partner | 23.07 | 7.09; 39.05 | 2.83 | **.01** |
|  | Education Middle | 10.60 | -3.51; 24.71 | 1.47 | .14 |
|  | Education Low | 9.50 | -7.86; 26.85 | 1.07 | .14 |
|  | Resilience | .55 | -.21; 1.31 | 1.42 | .16 |
|  | HLC Int. | 3.68 | -8.16; 15.51 | .61 | .54 |
|  | HLC Ext. | 4.87 | -1.87; 11.61 | 1.42 | .16 |
|  | PHQ | 4.68 | 2.76; 6.60 | 4.78 | **< .01** |
|  | Pain Current | .23 | .04; .43 | 2.32 | **.02** |
|  | Pain Desired | -.06 | -.43; .31 | -.30 | .76 |
|  | X²(11) = 31120.79, p < .001, Pseudo-R² (Cragg-Uhler) = .32, Pseudo-R² (McFadden) = .04,  AIC = 911.10, BIC = 944.16 | | | | |
| ADL | Intercept | -25.10 | -73.20; 23.00 | -1.02 | .31 |
|  | PHQ | 2.22 | 1.03; 3.41 | 3.66 | **< .01** |
|  | MoCA | 1.56 | -.44; 3.56 | 1.52 | .13 |
|  | X²(2) = 8483.23, p < .001, Pseudo-R² (Cragg-Uhler) = .20, Pseudo-R² (McFadden) = .03, AIC 0 786.94, BIC = 796.89 | | | | |
| Finances | Intercept | -8.10 | -58.02; 41.82 | -.32 | .75 |
|  | Age | -.21 | -.70; .28 | -.84 | .40 |
|  | Education Middle | -2.95 | -11.20; 5.39 | -.69 | .49 |
|  | Education Low | 5.46 | -4.44; 15.37 | 1.08 | .28 |
|  | HLC Int. | 8.02 | 1.22; 14.81 | 2.31 | **.02** |
|  | HLC Ext. | -2.50 | -6.47; 1.46 | -1.24 | .22 |
|  | PHQ | .92 | -.07; 1.91 | 1.83 | .07 |
|  | X²(6) = 4081.32, p = .03, Pseudo-R² (Cragg-Uhler) = .14, Pseudo-R² (McFadden) = .02, AIC = 811.10, BIC = 831.44 | | | | |
| Leisure | Intercept | -38.85 | -95.93; 18.24 | -1.33 | .19 |
|  | Resilience | -.21 | -.68; .26 | -.89 | .38 |
|  | PHQ | 2.65 | 1.48; 3.83 | 4.42 | **< .01** |
|  | Leisure Current | .11 | -.03; .25 | 1.50 | .14 |
|  | MoCA | 1.82 | .03; 3.61 | 1.99 | **.05** |
|  | X²(5) = 15270.45, p < .001, Pseudo-R² (Cragg-Uhler) = .38, Pseudo-R² (McFadden) = .06´5, AIC = 769.58, BIC = 787.00 | | | | |
| Family | Intercept | -23.65 | -71.09; 23.80 | -.98 | .33 |
|  | Age | .12 | -.22; .47 | .69 | .49 |
|  | Female | -5.98 | -11.18; -.78 | -2.26 | **.03** |
|  | Living With Partner | -2.87 | -9.42; 3.67 | -.86 | .39 |
|  | Education Middle | -2.63 | -8.51; 3.26 | -.87 | .38 |
|  | Education Low | 2.34 | -4.62; 9.29 | .66 | .51 |
|  | Resilience | -.08 | -.40; .25 | -.46 | .65 |
|  | HLC Int. | 4.06 | -.80; 6.91 | 1.61 | .11 |
|  | PHQ | 1.34 | .55; 2.13 | 3.33 | **< .01** |
|  | Family Desired | .09 | -.09; .27 | .95 | .34 |
|  | X²(9) = 3899.90, p < .001, Pseudo-R² (Cragg-Uhler) = .24, Pseudo-R² (McFadden) = .03, AIC = 747.19, BIC = 755.17 | | | | |
| Note: HLC = Health Locus of Control, PHQ = Patient Health Questionnaire, MoCA = Montreal Cognitive Assessment, ADL = Activities of Daily Living, CI = Confidence Interval | | | | | |


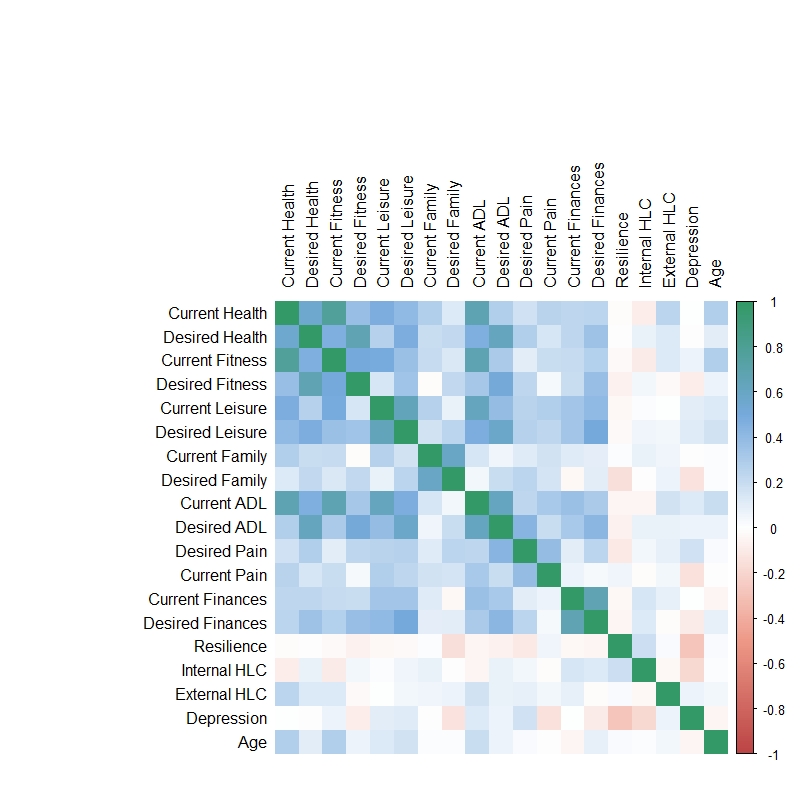


**Supplement Figure 1.** Correlation plot between the variables in the dataset based on spearman correlation.
Note: ADL = Activities of Daily Living, HLC = Health Locus of Control


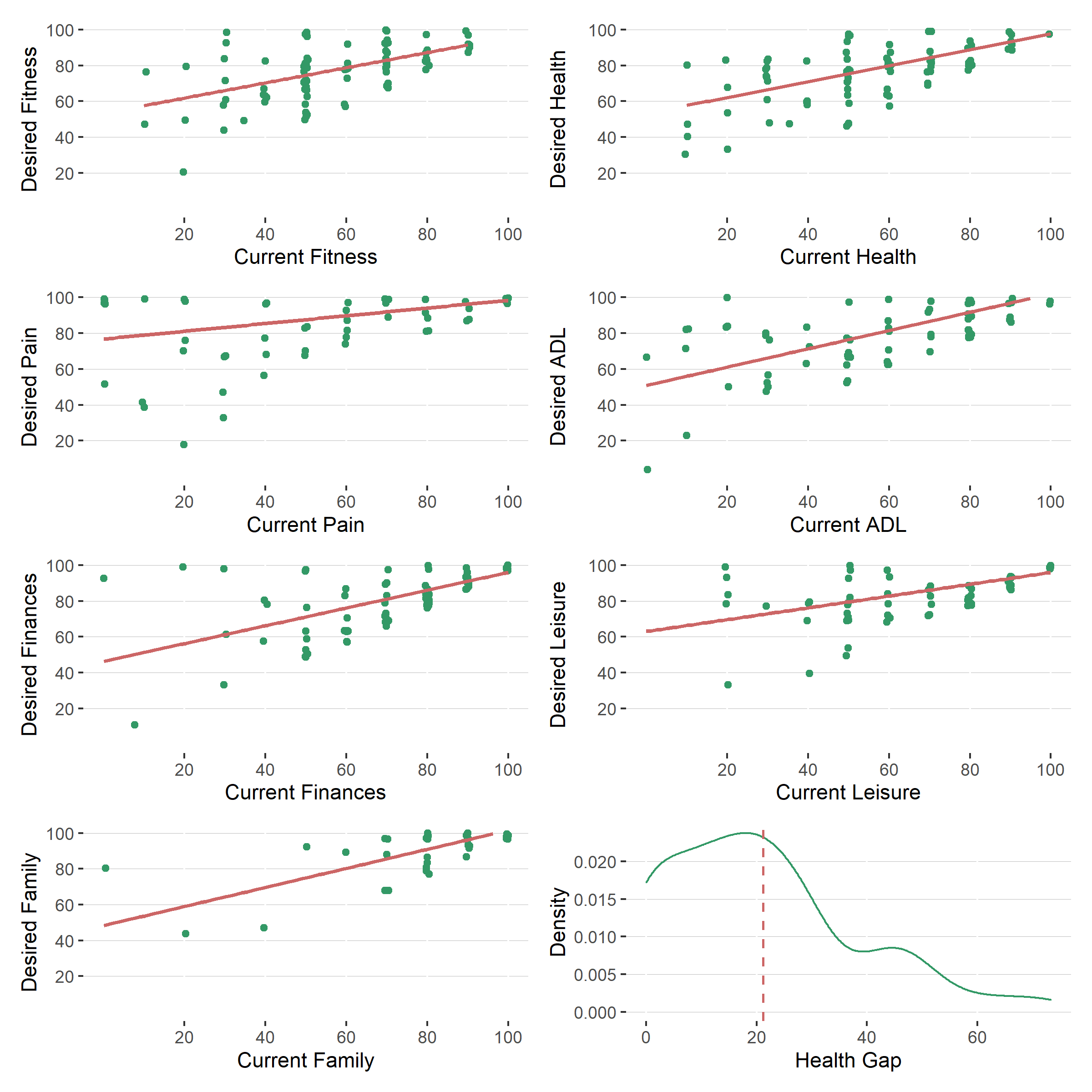


**Supplement Figure 2.** Relation of Current and Desired state for all domains and density of Health Gap.


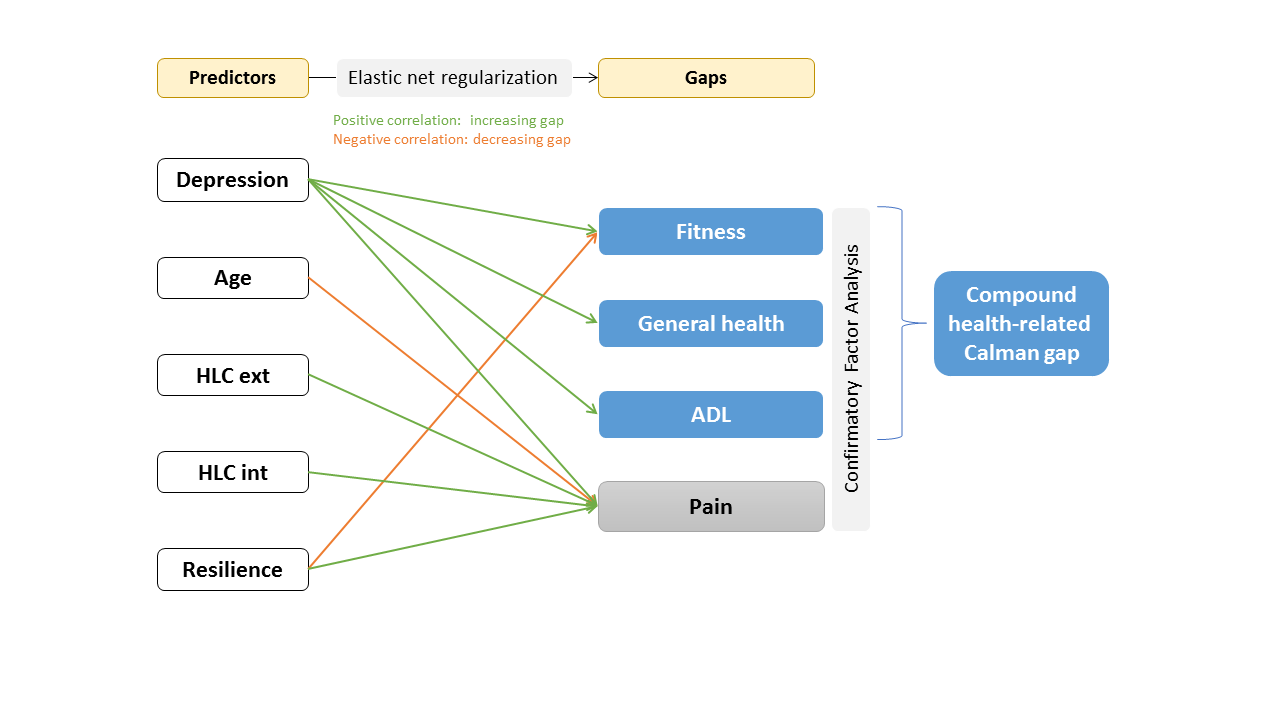

**Supplement Figure 3.** Summary of Calman gap predictors. Note: ADL = Activities of Daily Living, HLC = Health Locus of Control, ext = external, int = internal
